# Factors Associated with Overcrowded Emergency Rooms in Thailand: A Medical School Setting

**DOI:** 10.1155/2014/576259

**Published:** 2014-09-29

**Authors:** Arrug Wibulpolprasert, Yuwares Sittichanbuncha, Pungkava Sricharoen, Somporn Borwornsrisuk, Kittisak Sawanyawisuth

**Affiliations:** ^1^Department of Emergency Medicine, Faculty of Medicine, Ramathibodi Hospital, Mahidol University, Bangkok 10400, Thailand; ^2^Department of Medicine, Faculty of Medicine, Khon Kaen University, Khon Kaen 40002, Thailand; ^3^The Research and Training Center for Enhancing Quality of Life of Working-Age People, Khon Kaen University, Khon Kaen 40002, Thailand

## Abstract

*Background*. Overcrowding in the emergency department (ED) is a significant public health problem in the US, Europe, and Asia. Factors associated with prolonged length of stay in Thailand are still limited. *Methods*. This study was conducted at the ED, Ramathibodi Hospital, Mahidol University, Thailand, during July 2011. We selected 300 patients (5.77%) from a total of 5,202 who visited the ED during the study period by simple random sampling. Charts were retrospectively reviewed baseline characteristics, clinical factors, and duration of ED stay. Multivariate logistic regression analyses were performed to identify independent factors for an ED stay more than or equal to 8 hours. *Results*. We excluded 33 patients (11%) due to incomplete data or stroke fast track enrollment. In total, 267 patients were in the analysis and 53 patients (19.85%) had an ED visit time more than or equal to 8 hours. The number of rounds of blood testing and the type of insurance were associated with prolonged ED stay of more than or equal to 8 hours. *Conclusion*. ED physicians may need to consider appropriate investigations to shorten the length of stay in the ED.

## 1. Background 

Overcrowding of the emergency department (ED) is a significant public health problem in the United States of America (US), Europe, and Asia [1–4]. A survey in the US found that 94% of university hospitals and 91% of private hospitals had overcrowded EDs [1]. In addition, 59% of EDs in the US needed to use hallways to care for patients [5]. Overcrowding in the ED has been shown to increase mortality at 10 days by 1.34 times if the patients arrived at an overcrowded ED [6]. It also significantly delayed thrombolytic therapy in patients with acute myocardial infarction or in those patients with analgesic administration for acute severe pain [7, 8]. Patient satisfaction was significantly better if the patient's stayed in the ED less than 4 hours compared with patients who stayed more than 8 hours [9]. Long ED periods were associated with hospitalization particularly if the ED stay was more than 12 hours [10].

Factors that have been reported to be associated with overcrowded ED or prolonged ED stay are the numbers of patients, inappropriate ED visit [11], severity of patient conditions, laboratory investigations [12], interdepartmental consultations [13], and the types of health insurance [14]. Data concerning overcrowding in the ED have been predominately from the US [11–15]. In Thailand and other Asian countries, factors associated with overcrowding in the ED are still limited. This study aimed to identify factors related to ED overcrowding in an academic hospital in Thailand by using data from real clinical practice or a pragmatic study.

## 2. Methods

The study was conducted in the ED at Ramathibodi Hospital, Mahidol University. Patients who visited the ED during July, 2011, were enrolled. We excluded patients who were in fast track treatment for acute coronary syndrome, stroke, or a pediatric hematology/oncology sepsis fast track. Patients who left without being seen or had incomplete data were also excluded. Medical records of eligible patients were reviewed.

The following clinical data were retrospectively collected from medical records: time of ED stay and discharge status from the ED and also baseline characteristics, comorbid diseases, type of insurance, presenting time, patient category or patient severity, laboratory investigations, and interdepartmental consultation. The ED stay time was defined by the discharge time from the ED subtracted by the registered time at the ED. Discharge status from the ED was categorized as went home, referred to other hospitals, or admitted.

There are four categories of health insurance in Thailand: self-payment, government insurance, social security insurance, and universal coverage. Patients can present to the ED on their own, by ambulance, and by referral. Patient category (severity) was defined by a nurse at the time of presentation. Triage in the ED was the chief complaint which was based and categorized into four levels: Level 1, patients with critical conditions who needed immediate attention; Level 2, patients with acute emergency illness; Level 3, patients with acute illness, but not severe; Level 4, patients without emergency conditions who did not require emergency care. The number of consultations was defined by the number of interdepartmental consultations per patient. The number of rounds of blood testing was defined by how many times physicians ordered blood tests for each patient.

## 3. Statistical Analyses

The sample size of study population was calculated from a pilot study which was done previously at the ED of Ramathibodi Hospital. The required sample size was 78 patients to have confidence of 95% and power of 80% by using sample size estimation for correlation coefficients (based on the *r* value from the pilot study of −0.2817). Eligible patients were selected by a simple random sampling method by using a random number generator (http://stattrek.com/sampling/simple-random-sampling.aspx). Assuming missing data and loss of medical records, we randomly selected the total of 300 subjects for the study.

Patients were categorized into two groups: ED stay less than 8 hours and ED stay more than or equal to 8 hours. Baseline and clinical characteristics of both groups were compared using descriptive statistics. Wilcoxon rank-sum or Student's *t*-test and Fisher's exact tests or Chi-square test were applied to compare the differences in numbers and proportions between the two groups, respectively.

Univariate logistic regression analyses were applied to calculate the crude odds ratios of individual variables for an ED stay of more than or equal to 8 hours. All factors with *P* value less than 0.1 by univariate logistic analyses were included in subsequent multivariate logistic regression analyses. Analytical results by multivariate logistic regression were presented as crude odds ratios (OR), adjusted OR, and 95% CI. All data analyses were performed with STATA software (College station, Texas, USA).

## 4. Results

There were 5,202 patients who visited the ED during the study period. Of those, 300 patients (5.77%) were randomly selected. Thirty-three patients were excluded due to having no medical records (21 patients), stroke fast track (5 patients), and incomplete ED visit time (3 patients) and being left before being seen (1 patient). In total, 267 patients were included in the analysis and 53 patients (19.85%) had an ED visit time of more than or equal to 8 hours (Table 1).

Patients who had an ED visit time of more than or equal to 8 hours were of significantly older age (67 versus 42.5 years) than patients who had an ED visit time of less than 8 hours (Table 1). The older age group included a higher proportion of patients requiring consultation (75.47% versus 39.72%), more severe patients (7.55% versus 1.87% in category 1), a higher proportion of patients with 2 comorbid diseases (49.06% versus 18.22%), consultation of more than or equal to 2 departments (3.77% versus 0.47%), admission to a hospital ward (28.30% versus 10.28%), and subsequent laboratory investigations which included blood testing, general X-ray, computed tomography, and ultrasonography (Table 1).

There were two factors which were found to be statistically significant by multivariate logistic regression analyses. The number of rounds of blood testing and the type of insurance were associated with prolonged ED stay of more than or equal to 8 hours. The adjusted ORs of both factors are shown in Table 2.

## 5. Discussion

After adjusting or controlling for confounding factors, two independent factors were found to be statistically significant for prolonged ED stay by multivariate logistic regression analyses, namely, rounds of blood testing and the type of health insurance.

Repeated blood testing was an independent factor for prolonged ED stay particularly if the attending physicians ordered more than two rounds of blood testing. The adjusted ORs and coefficient values were dramatically higher in those patients with more than two rounds of blood testing (Table 2). Previous studies have shown that the number of rounds of blood testing significantly increased ED stay time [12, 13, 16]. The Ramathibodi hospital, as well as other academic hospitals in Thailand, has a “bed block” problem which results in increased ED length of stay. Therefore, the longer the time patients wait in the ED, the more the laboratory tests requested. Reasons for multiple rounds of blood testing may be due to follow-up or new laboratory tests. Whether the multiple rounds of blood testing are appropriate or not was not investigated in this study. If inappropriate laboratory testing is the main problem, physicians may need to consider a more appropriate blood testing protocol hence shortening the time patients spend in the ED.

Government insurance was another independent factor. This factor may not apply for hospitals in other parts of Thailand or other countries. Most public hospitals in Thailand have unlimited extra inpatient beds along corridors. This policy transfers the overcrowding problem from the ED to inpatient departments. In the US, patients without health insurance spend more time at the ER [17–19]. The difference between these findings from results of the present study may be due to different health insurance policies/status.

Thai citizens are registered in three types of government-based health insurance schemes: government insurance, social security insurance, and universal coverage. Government insurance is granted to every government officer and their next of kin and can be used in any public hospital. For the social security and universal coverage systems, patients must register at one hospital and the hospital has an obligation/responsibility to cover the cost of any illness or accident of their registered patients. In the case of an emergency, patients can go to any hospital and transfer back to their registered hospital after stabilization. Since almost every public hospital throughout Bangkok has a bed block problem, most hospitals, including Ramathibodi hospital, have policies to allocate inpatient beds to their registered patients as a first priority. When a bed block problem occurs and patients are referred to other hospitals, the success rate was almost 100% when the patients were referred to their registered hospitals. This is in contrast to patients with government insurance and who are not registered to any specific hospital. Therefore, physicians need to keep these patients until they are admitted or discharged from the ED. In our study, all 9 patients who were transferred to other hospitals were patients without government insurance (Table 1). This bed block situation is considered as the important issue that leads to overcrowding in the ED which is similar to the access block issue previously reported in Australia [20]. The number of patients who require admission in the group with an ED visit time of more than or equal to 8 hours had a higher admission rate than the group with ED visit time less than 8 hours (28.30% versus 10.28%). These patients needed to stay in either the observation area or the ED hallways which resulted in long ED stays. Those with longer ED stay were also admitted as the first priority.

To reduce ED stay, ED physicians should consider more appropriate blood testing and ultrasonography investigation protocols. Protocols need to be developed for ER physicians to streamline testing [21]. These ED protocols may have universal applicability globally. The insurance issue seems to be out of the ED physicians' jurisdiction and it may be unique to Thailand hospitals.

The limitations of this present study include retrospective data collection and the number of patients sampled. Some data may be missing but the time of ED stay was accurately recorded by ED nurses. The study population accounted for 5.77% of 5,202 patients. Due to high numbers of ED patients, we randomly enrolled patients for about 5% of surveyed population. Despite the small sample size of the population examined, significant factors were found by statistical analyses. Another major limitation of this study is that physician-patient contact time during an ED visit was not recorded due to retrospective data collection. One factor that may affect the ED stay is sickness rate of staff, physicians, or consultants. These data were also not available. Even though the type of insurance in this study was significantly associated with the duration of ED, it may not be applicable universally.

In conclusion, ED physicians may need to consider appropriate investigations to shorten the length of stay in the ED.

## Figures and Tables

**Table 1:** Characteristics of patients at the emergency department (ED) categorized by time spent at the ED.

Factors	<8 hours *N* = 214	≥8 hours *N* = 58	*P* value
Male gender	88 (41.12)	27 (50.94)	0.196
Median age, years	42.5 (1–91)	67 (1–94)	<0.001
Types of health insurance			<0.001
Self-payment	94 (43.93)	9 (16.98)	
Government insurance	85 (39.72)	40 (75.47)	
Types of presenting to ER			<0.001
On their own	199 (92.99)	39 (73.58)	
Consultation	10 (4.67)	13 (24.53)	
Day presenting: Sunday	29 (13.55)	11 (20.75)	0.710
Times of presenting			0.515
08:00–16:00	74 (34.58)	18 (33.96)	
16:01–24:00	77 (35.98)	23 (43.40)	
00.01:–07:59	63 (29.44)	12 (22.64)	
Patient triage categories			0.018
1	4 (1.87)	4 (7.55)	
2	82 (38.32)	28 (52.83)	
3	117 (54.67)	20 (37.74)	
4	11 (5.14)	1 (1.89)	
Comorbid diseases			<0.001
None	111 (51.87)	13 (24.53)	
1	64 (29.91)	14 (26.42)	
2	39 (18.22)	26 (49.06)	
Consultation			<0.001
None	183 (85.51)	23 (43.40)	
1 department	30 (14.02)	28 (52.83)	
≥2 departments	1 (0.47)	2 (3.77)	
Types of discharge			0.003
Home	185 (86.45)	36 (67.22)	
Referred	7 (3.27)	2 (3.77)	
Admitted	22 (10.28)	15 (28.30)	
Blood testing			<0.001
None	134 (62.62)	3 (5.66)	
1 round	65 (30.37)	11 (20.75)	
≥2 rounds	15 (7.01)	39 (73.58)	
General X-ray	56 (26.17)	40 (75.47)	<0.001
Computed tomography	7 (3.27)	8 (15.09)	0.003
Ultrasonography	1 (0.47)	4 (7.55)	0.006

*Note.* Patient category: disease severity; see details in text; data presented as numbers (percentage) unless indicated otherwise; *P* values were obtained by Wilcoxon rank-sum or Student's *t*-test and Fisher's exact tests or Chi-square test where appropriated; total numbers of patients in both groups may not be equal to 214 and 58, respectively, due to missing data.

**Table 2:** Significant factors associated with prolonged stay more than or equal to 8 hours at the emergency department (ED) by univariate and multivariate logistic analyses.

Factors	Unadjusted ORs (95% CI)	Adjusted ORs (95% CI)
Blood testing		
None	1	1
1 round	7.56 (2.04, 28.23)	7.67 (2.03, 28.95)
≥2 rounds	116.13 (31.97, 421.83)	114.43 (30.30, 432.08)
Health insurance		
Self-payment	1	1
Government insurance	4.91 (2.25, 10.72)	4.07 (1.51, 10.96)

*Note.* ORs: odds ratios; 95% CI: 95% confidence interval; model adjusted for age, presentation type of presenting to ED, patient severity or category, type of health insurance, comorbid diseases, blood testing, consultation, general X-ray, computed tomography, ultrasonography, and type of discharge.
